# Micronutrient supplement intakes among collegiate and masters athletes: A cross-sectional study

**DOI:** 10.3389/fspor.2023.854442

**Published:** 2023-04-06

**Authors:** Quentin Z. Nichols, Rohit Ramadoss, Joseph R. Stanzione, Stella L. Volpe

**Affiliations:** ^1^ Department of Human Nutrition, Foods, and Exercise, Virginia Polytechnic Institute and State University (Virginia Tech), Blacksburg, VA, United States; ^2^ Nutrition & Scientific Affairs, Worldwide Sport Nutritional Supplements, Bohemia, NY, United States

**Keywords:** collegiate athletes, dietary intake, masters athletes, micronutrients, supplementation

## Abstract

**Objective:**

In our cross-sectional study, we evaluated micronutrient supplementation intake among Collegiate and Masters Athletes.

**Methods:**

We conducted a cross-sectional study to assess micronutrient supplementation consumption in Collegiate and Masters Athletes, comparing sex and sport classification within each respective group. Micronutrient supplement consumption data were measured using a Food Frequency Questionnaire. A two-way analysis of variance was used to explore the differences among Collegiate and Masters Athletes' supplement intakes of the following vitamins and minerals: vitamins A, B_6_, B_12_, C, E, D, and calcium, folate, iron, magnesium niacin, riboflavin, selenium, thiamine, and zinc. When significant differences were found, a Bonferroni *post hoc* test was performed to identify specific group differences. The significance level was set *a priori* at *p* < 0.05.

**Results:**

A total of 198 athletes (105 females and 93 males) were included in the study. Participants were 36.16 ± 12.33 years of age. Collegiate male athletes had significantly greater vitamin A [1,090.51 ± 154.72 vs. 473.93 ± 233.18 mg retinol activity equivalents (RAE)/day] (*p* < 0.036), folate [337.14 ± 44.79 vs. 148.67 ± 67.50 mcg dietary folate equivalents (DFE)/day] (*p* < 0.027), and magnesium (65.35 ± 8.28 vs. 31.28 ± 12.48 mg/day) (*p* < 0.031) intakes compared to Collegiate female athletes. Collegiate CrossFit Athletes (940.71 ± 157.54 mg/day) had a significantly greater vitamin C intake compared to Collegiate General Athletes (156.34 ± 67.79 mg/day) (*p* < 0.005), Collegiate Triathletes (88.57 ± 148.53 mg/day) (*p* < 0.027), Collegiate Resistance Training Athletes (74.28 ± 143.81 mg/day) (*p* < 0.020), and Collegiate Powerlifters (175.71 ± 128.63 mg/day) (*p* < 0.044). Masters females had significantly greater calcium intakes compared to Masters males (494.09 ± 65.73 vs.187.89 ± 77.23 mg/day, respectively) (*p* < 0.002). Collegiate Runners (41.35 ± 6.53 mg/day) had a significantly greater iron intake compared to Collegiate Powerlifters (4.50 ± 6.53 mg/day) (*p* < 0.024). Masters Swimmers (61.43 ± 12.10 mg/day) had significantly greater iron intakes compared to Masters General Athletes (13.97 ± 3.56 mg/day) (*p* < 0.014), Masters Runners (17.74 ± 2.32 mg/day) (*p* < 0.03), Masters Triathletes (11.95 ± 3.73 mg/day) (*p* < 0.008), Masters CrossFit Athletes (15.93 ± 5.36 mg/day) (*p* < 0.043), Masters Rowers (9.10 ± 3.36 mg/day) (*p* < 0.003), and Masters Cyclists (1.71 ± 9.88 mg/day) (*p* < 0.011). Masters Powerlifters (47.14 ± 9.65 mg/day) had significantly greater zinc intakes compared to Masters General Athletes (9.57 ± 2.84 mg/day) (*p* < 0.015), Masters Runners (10.67 ± 1.85 mg/day) (*p* < 0.017), Masters Triathletes (10.24 ± 2.98 mg/day) (*p* < 0.020), Masters Rowers (9.33 ± 2.68 mg/day) (*p* < 0.013), and Masters Cyclists (1.43 ± 7.88 mg/day) (*p* < 0.019). There were no other significant differences among the other micronutrient supplement intakes between the sexes or among the sport classification.

**Conclusion:**

We reported significant differences among female and male Collegiate and Masters Athletes. Additionally, we reported significant differences among Collegiate and Masters Athletes sport classifications. Further research should examine both dietary and micronutrient supplement intake among Collegiate and Masters Athletes to examine the extent that athletes exceed the Recommended Dietary Allowances (RDA), and the potential effects on health and performance.

## 1. Introduction

Micronutrients play a vital role in the maintenance of tissue function and are required for a number of metabolic reactions, including energy production. During physical activity, micronutrients are involved in roles such as muscle contraction, oxygen transport, and the metabolism of carbohydrates, protein and fat (1–3). Physical activity increases the utilization of micronutrients by increasing energy metabolism production, increased demand from exercising tissue, and losses from sweat, urine, and feces (2, 4, 5). Physical activity, coupled with insufficient energy intake, may lead to vitamin and mineral deficiencies (3), which can lead to impaired athletic performance and health. Athletes may take a vitamin and mineral supplement, or take individual vitamins and minerals based on the potential performance benefit of the supplement (6). Further, athletes may take micronutrient supplements as a result of inadequate dietary intake, dietary restrictions, or consuming vegan/vegetarian diets (7, 8). A Joint Position Stand published by the American College of Sports Medicine, the Academy of Nutrition and Dietetics, and the Dietitians of Canada (3) concluded that micronutrient supplementation may be required if athletes restrict their energy intake, remove food groups from their diets, or consume high carbohydrate diets, and/or low micronutrient density diets. Endurance athletes such as runners, cyclists, and triathletes may be most at risk for micronutrient deficiency because of low energy consumption, coupled with longer physical activity duration and increased sweat rate (9, 10). Wirnitzer et al. (11) reported that 51% of endurance runners regularly consumed a vitamin and mineral supplement.

Previous researchers have reported that 80% of collegiate athletes use supplements, one of the most common being multi-vitamin and multi-mineral supplements (6, 12–14). Parnell et al. (15) reported that college-age athletes (19–25 years of age) who took individual vitamin, mineral or multi-vitamin/multi-mineral supplements thought their athletic performance would improve (15).

Masters Athletes are usually between 35 and 50 years of age and compete in organized sports (16, 17); however, they can be younger than 35 years of age depending on the specific sport's requirement (17). Masters Athletes may be more likely to take micronutrient supplements because of the various challenges that occur with aging and recovery (18–20).

The prevalence of supplement use among female athletes is similar to male athletes, although their reasons for use differ (6, 13, 21, 22). Bailey et al. (21) reported that supplement use among 2,066 females, ≥20 years of age, was mainly to improve bone health and to increase energy. Similarly, Barrack et al. (22) reported that, among 557 Collegiate Athletes (259 females, 298 males), the use of a micronutrient supplement was highest among female athletes wanting to improve health. Senekal et al. (23) reported that, regardless of age, younger men (21–30 years of age) and older males (≥45 years of age) took micronutrient supplements for performance improvements and to maintain overall health.

Micronutrient supplementation has had widespread use, regardless of age, sex and sport level (6, 13, 23, 24, 25). The purpose of our study was to compare the differences in vitamin and mineral supplementation between the sexes and among various sport classifications in Collegiate Athletes and Masters Athletes.

## 2. Materials and methods

### 2.1. Study design and participants

We conducted a cross-sectional study that included Collegiate and Masters Athletes from multiple sporting backgrounds. Participant eligibility was assessed using an online survey. Participants qualified based on the following inclusion criteria: non-smokers, Collegiate or Masters Athletes who exercised at least two days per week. Exclusion criteria included: sedentary individuals (defined as persons who exercised less than two days a week), individuals diagnosed with chronic disease (e.g., cardiovascular disease, hypertension) without written permission from their physician, individuals taking medications without written permission from their physician, adults unable to consent, individuals less than 18 years of age, and pregnant. Although Masters Athletes' age can vary among different sports, we defined Masters Athletes as being 26 years of age and older, based on the fact that some sports consider ≥21 years of age as a Masters Athlete, while others consider ≥35 years of age as a Masters Athlete. The sport classifications used in our study were General Athletes, Runners, Triathletes, Rowers, Cyclists, CrossFit Athletes, Resistance Training Athletes, Powerlifters, Soccer Athletes, Track Athletes, Tennis Athletes, Football Athletes, Ice Hockey Athletes, Field Hockey Athletes, and Swimmers. General Athletes were defined as athletes who engaged in multiple sports. Athlete levels were characterized as recreational, club, varsity (applies only to Collegiate Athletes) and professional (one Masters Triathlete). The requirements previously mentioned to classify each athlete aligns with the Tier 1 and 2 criteria for participation classification established by McKay et al. (26). Each potential participant was asked to complete an online survey *via* the REDCap™ software. Drexel University's Institutional Review Board approved all study procedures and protocols prior to any data collection.

### 2.2. Measurements

Participants who qualified were scheduled to have their measurements taken at the Nutrition Sciences Laboratory at Drexel University. Body weight and height were measured twice on a calibrated scale and stadiometer, respectively. Body weight and height were measured twice to ensure accuracy. Waist circumference was measured one inch above the umbilicus, three times to ensure accuracy, using a soft measuring tape. Afterwards, the participants completed the Block 2005 Food Frequency Questionnaire (FFQ). The Block 2005 FFQ is a self-administered FFQ that took about 30–40 min to complete. The Block 2005 FFQ questions about supplements are limited only to single vitamin, mineral, and multivitamin and multimineral use. Additionally, there were no questions about supplements that contained vitamins and minerals such as protein powders, sport gels/powders, pre-workout supplements, and fruit and vegetable powders. The micronutrient supplementation in which we focused for this study were: vitamins A, B_6_, B_12_, C, E, D, and calcium, folate, iron, magnesium niacin, riboflavin, selenium, thiamine, and zinc. The Block FFQs were sent to NutritionQuest (Berkeley, CA) for analyses.

### 2.3. Statistical analyses

Statistical analyses were performed using using SPSS Statistics (Version 26.0. Armonk, NY, IBM Corp). A two-way analysis of variance (ANOVA) was used to compare the following vitamin and mineral supplement intakes: vitamins A, B_6_, B_12_, C, E, D, and calcium, folate, iron, magnesium niacin, riboflavin, selenium, thiamine, and zinc among Collegiate and Masters Athlete sex and sport classifications. When significant differences were found, a Bonferroni *post hoc* test was performed to identify specific group differences between sex and among sport classifications among Collegiate and Masters athletes. The significance level was set *a priori* at *p* < 0.05, no additional corrections were conducted for type II errors. The descriptive characteristics of Collegiate and Masters Athletes groups and subgroups was reported as mean ± standard deviation (SD). Due to uneven numbers of participants among sport classifications, vitamin and mineral intake were reported as estimated marginal means ± standard error (SE). Normality of the data was evaluated with visual inspections of histograms and normal probability plots and the Kruskal–Wallis test.

## 3. Results

### 3.1. Descriptive statistics

Our study included 365 adults, 18 years of age or older, who were Collegiate and Masters Athletes. A total of 198 athletes (105 females and 93 males) who used at least one micronutrient supplement were included in the final statistical analyses. Table 1 outlines the descriptive characteristics of the 198 athletes who submitted complete data regarding micronutrient supplement use.

**Table 1:** Descriptive characteristics of collegiate and masters athletes.

Characteristics	Collegiate Athletes			Masters Athletes		
	Females (*n* = 17)	Males (*n* = 27)	Combined (*n* = 44)	Females (*n* = 88)	Males (*n* = 66)	Combined (*n* = 154)
Age (years)	22.76 ± 1.85	21.63 ± 2.09	22.07 ± 2.06	41.31 ± 10.50	38.70 ± 11.69	40.19 ± 11.05
Height (cm)	165.82 ± 6.34	177.67 ± 7.16	173.09 ± 8.94	165.21 ± 6.80	179.09 ± 7.46	171.16 ± 9.87
Weight (kg)	59.17 ± 9.19	79.29 ± 9.04	71.52 ± 13.38	66.48 ± 11.20	81.38 ± 11.18	72.86 ± 13.38
BMI (kg/m^2^)	21.44 ± 2.49	25.12 ± 2.59	23.69 ± 3.11	24.34 ± 3.75	25.31 ± 2.56	24.75 ± 3.32

cm, centimeters; kg, kilograms; BMI, body mass index; kg, kilograms; m^2^, meters squared.

Values represent Mean ± Standard Deviation.

### 3.2. Micronutrient supplement use

There were significant differences in supplement use among Collegiate female and male athletes (Table 2). Collegiate male athletes had significantly greater vitamin A [1,090.51 ± 154.72 vs. 473.93 ± 233.18 mg retinol activity equivalents (RAE)/day] (*p* < 0.036), folate [337.14 ± 44.79 vs. 148.67 ± 67.50 mcg dietary folate equivalents (DFE)/day] (*p* < 0.027), and magnesium (65.35 ± 8.28 vs. 31.28 ± 12.48 mg/day) (*p* < 0.031) intakes compared to Collegiate female athletes, respectively. There were significant differences among sport classification supplement use (Table 3) among Collegiate Athletes. Collegiate CrossFit Athletes (940.71 ± 157.54 mg/day) had a significantly greater vitamin C intake compared to Collegiate General Athletes (156.34 ± 67.79 mg/day) (*p* < 0.005), Collegiate Triathletes (88.57 ± 148.53 mg/day) (*p* < 0.027), Collegiate Resistance Training Athletes (74.28 ± 143.81 mg/day) (*p* < 0.020), and Collegiate Powerlifters (175.71 ± 128.63 mg/day) (*p* < 0.044). Collegiate Runners (41.35 ± 6.53 mg/day) had a significantly greater iron intake compared to Collegiate Powerlifters (4.50 ± 6.53 mg/day) (*p* < 0.024). There were significant differences among Masters female and male athletes' supplement use (Table 4). Masters female athletes had significantly greater calcium intakes compared to Masters male athletes (494.09 ± 65.73 vs.187.89 ± 77.23 mg/day, respectively) (*p* < 0.002). There were also significant differences in supplement use based on sport classification among Masters Athletes (Table 5). Masters Swimmers (61.43 ± 12.10 mg/day) had significantly greater iron intakes compared to Masters General Athletes (13.97 ± 3.56 mg/day) (*p* < 0.014), Masters Runners (17.74 ± 2.32 mg/day) (*p* < 0.03), Masters Triathletes (11.95 ± 3.73 mg/day)(*p* < 0.008), Masters CrossFit Athletes (15.93 ± 5.36 mg/day) (*p* < 0.043), Masters Rowers (9.10 ± 3.36 mg/day) (*p* < 0.003), and Masters Cyclists (1.71 ± 9.88 mg/day) (*p* < 0.011). Masters Powerlifters (47.14 ± 9.65 mg/day) had significantly greater zinc intakes compared to Masters General Athletes (9.57 ± 2.84 mg/day) (*p* < 0.015), Masters Runners (10.67 ± 1.85 mg/day) (*p* < 0.017), Masters Triathletes (10.24 ± 2.98 mg/day) (*p* < 0.020), Masters Rowers (9.33 ± 2.68 mg/day) (*p* < 0.013), and Masters Cyclists (1.43 ± 7.88 mg/day) (*p* < 0.019) (Table 5). There were no other significant differences between the Collegiate and Masters groups.

**Table 2:** Micronutrient supplementation among female and male collegiate athletes.

Micronutrient Supplement	Collegiate Athletes		
	Females	Males	Combined
Vitamin A (mcg RAE)^a^	473.93 ± 233.18	1,090.51 ± 154.72*	859.30 ± 130.37
Vitamin C (mg)^a^	313.44 ± 89.54	309.76 ± 59.41	311.14 ± 50.06
Vitamin D (mcg)^a^	5.71 ± 1.45	7.60 ± 0.96	6.89 ± 0.81
Vitamin E (mg d-alpha tocopherol)^a^	7.90 ± 10.18	12.09 ± 6.75	10.57 ± 5.69
B-1 (Thiamin) (mg)^a^	1.05 ± 0.90	2.37 ± 0.59	1.88 ± 0.50
B-6 (mg)^a^	0.92 ± 0.51	2.02 ± 0.33	1.61 ± 0.28
B-12 (mcg)^a^	2.58 ± 1.30	5.61 ± 0.86	4.47 ± 0.73
Folate (mcg DFE)^a^	148.67 ± 67.50	337.14 ± 44.79**	266.46 ± 37.74
Niacin (mg)^a^	12.14 ± 9.17	27.10 ± 6.08	21.49 ± 5.13
Riboflavin (mg)^a^	1.12 ± 0.90	2.50 ± 0.60	1.98 ± 0.50
Calcium (mg)^a^	543.99 ± 116.39	271.90 ± 77.23	373.93 ± 65.07
Iron (mg)^a^	10.61 ± 4.54	12.89 ± 3.01	12.04 ± 2.54
Magnesium (mg)^a^	31.28 ± 12.48	65.35 ± 8.28***	52.57 ± 6.97
Selenium (mcg)^a^	6.25 ± 4.47	16.12 ± 2.96	12.42 ± 2.49
Zinc (mcg)^a^	7.60 ± 4.05	15.01 ± 2.69	12.23 ± 2.26

mcg, micrograms; RAE, retinol activity equivalents; mg, milligrams; DFE, dietary folate equivalents.

^a^ Data are presented as estimated marginal means ± standard error.

* Collegiate Male Athletes (1,090.51 ± 154.72 mcg RAE/day) had significantly greater vitamin A intakes compared to Collegiate Female Athletes (473.93 ± 233.18 mcg RAE/day) (*p* = 0.036).

** Collegiate Male Athletes (337.14 ± 44.79 mcg DFE/day) had significantly greater folate intakes compared to Collegiate Female Athletes (148.67 ± 67.50 mcg DFE/day) (*p* = 0.027).

*** Collegiate Male Athletes (65.35 ± 8.28 mg/day) had significantly greater magnesium intakes compared to Collegiate Female Athletes (31.28 ± 12.48 mg/day) (*p* = 0.031).

**Table 3:** Collegiate athletes sports classification micronutrient supplementation.

Micronutrient Supplementation	Collegiate Athletes										
	Sport Classification										
	GA (*n* = 15)	RN (*n* = 4)^b^	TA (*n* = 4)	CF (*n* = 3)	RW (*n* = 2)^b^	RT (*n* = 5)	PL (*n* = 4)^b^	SC (*n* = 3)^b^	FB (*n* = 1)^b^	TK (*n* = 2)	TN (*n* = 1)^b^
Vitamin A (mcg RAE)^a^	1,142.26 ± 176.54	1,136.25 ± 334.97	432.85 ± 386.80	757.50 ± 410.26	1,298.57 ± 473.73	973.93 ± 374.51	378.75 ± 334.97	1,292.14 ± 386.80	432.86 ± 669.95	757.50 ± 473.73	1,082.14 ± 669.95
Vitamin C (mg)^a^	156.34 ± 67.79	214. 64 ± 128.63	88.57 ± 148.53	940.71 ± 157.54*	265.71 ± 181.91	74.28 ± 143.81	175.71 ± 128.63	325.24 ± 148.53	302.86 ± 257.92	280.00 ± 182.91	614.29 ± 257.26
Vitamin D (mcg)^a^	8.74 ± 1.09	10.0 ± 2.08	5.35 ± 2.40	7.50 ± 2.55	8.57 ± 2.94	7.14 ± 2.32	3.75 ± 2.08	3.33 ± 2.40	2.85 ± 4.16	6.78 ± 2.94	10.71 ± 4.16
Vitamin E (mg d-alpha tocopherol)^a^	19.43 ± 7.70	13.71 ± 14.62	4.81 ± 16.89	6.75 ± 17.91	11.57 ± 20.68	8.68 ± 16.35	6.96 ± 14.62	9.72 ± 16.89	3.86 ± 29.25	9.62 ± 20.68	24.00 ± 29.25
B-1 (mg)^a^	2.00 ± 0.68	2.91 ± 1.29	0.90 ± 1.49	0.75 ± 1.58	1.28 ± 1.82	0.96 ± 1.44	2.16 ± 1.29	1.50 ± 1.49	0.42 ± 2.58	2.17 ± 1.82	8.21 ± 2.58
B-6 (mg)^a^	1.94 ± 0.38	2.39 ± 0.73	0.81 ± 0.84	1.00 ± 0.90	1.71 ± 1.03	1.28 ± 0.82	1.39 ± 0.73	1.21 ± 0.84	0.57 ± 1.46	1.71 ± 1.03	5.00 ± 1.46
B-12 (mcg)^a^	5.57 ± 0.99	6.64 ± 1.87	2.28 ± 2.16	3.00 ± 2.30	5.14 ± 2.65	3.85 ± 2.10	3.64 ± 1.87	2.78 ± 2.16	1.71 ± 3.75	4.71 ± 2.65	12.86 ± 3.75
Folate (mcg DFE)^a^	336.50 ± 51.11	371.42 ± 96.97	133.33 ± 111.97	200.00 ± 118.76	342.85 ± 137.14	257.14 ± 108.42	171.43 ± 96.97	323.81 ± 111.97	114.29 ± 193.94	257.14 ± 137.14	571.43 ± 193.94
Niacin (mg)^a^	23.81 ± 6.94	32.85 ± 13.18	10.47 ± 15.21	10.00 ± 15.21	17.14 ± 16.14	12.85 ± 14.73	22.85 ± 13.18	16.76 ± 15.21	5.71 ± 26.36	24.28 ± 18.64	85.71 ± 26.36
Riboflavin (mg)^a^	2.15 ± 0.68	3.06 ± 1.30	0.96 ± 1.50	0.85 ± 1.59	1.45 ± 1.84	1.09 ± 1.45	2.21 ± 1.30	1.54 ± 1.50	0.48 ± 2.60	2.27 ± 1.84	8.36 ± 2.60
Calcium (mg)^a^	284.12 ± 88.12	501.42 ± 167.21	735.23 ± 193.08	620.00 ± 204.79	205.71 ± 236.47	297.14 ± 186.49	60.00 ± 167.21	148.57 ± 193.08	68.57 ± 334.42	477.14 ± 236.47	171.43 ± 334.42
Iron (mg)^a^	14.60 ± 3.44	41.35 ± 6.53**	5.14 ± 7.54	9.00 ± 8.00	15.43 ± 9.23	11.57 ± 7.30	4.50 ± 6.53	14.76 ± 7.54	5.14 ± 13.06	9.00 ± 9.23	12.86 ± 13.06
Magnesium (mg)^a^	75.39 ± 9.44	75.00 ± 17.92	28.57 ± 20.70	50.00 ± 21.70	85.71 ± 24.35	64.28 ± 20.04	25.00 ± 17.92	19.04 ± 20.07	28.57 ± 35.86	50.0 ± 24.35	71.43 ± 35.85
Selenium (mcg)^a^	15.08 ± 3.38	15.00 ± 6.42	5.71 ± 7.41	10.00 ± 7.86	17.14 ± 9.08	12.85 ± 7.18	5.00 ± 6.42	34.28 ± 7.41	5.71 ± 12.84	10.00 ± 9.08	14.29 ± 12.84
Zinc (mcg)^a^	14.08 ± 3.07	11.25 ± 5.82	4.28 ± 6.72	7.50 ± 7.13	12.85 ± 8.24	23.03 ± 6.51	34.10 ± 5.82	9.71 ± 6.72	4.29 ± 11.65	7.50 ± 8.42	10.71 ± 11.65

GA, general athlete; RN, runner; TA, triathlete; CF, crossFit; RW, rower; RT, resistance training; PL, powerlifter; SC, soccer; FB, football; TK, track, TN, tennis; mcg, microgram; mg, milligram; RAE, retinol activity equivalents; DFE, dietary folate equivalents.

^a^ Data are presented as estimated marginal means ± standard error.

^b^ Based on modified population marginal means.

* Collegiate CrossFit Athletes (940.71 ± 157.94 mg/day) had a significantly greater vitamin C intake compared to Collegiate General Athletes (158.73 ± 67.96 mg/day) (*p* = 0.006), Collegiate Triathletes (88.57 ± 148.91 mg/day) (*p* = 0.03), Collegiate Resistance Training Athletes (74.28 ± 144.18 mg/day) (*p* = 0.021), and Collegiate Powerlifters (175.71 ± 128.63 mg/day) (*p* = 0.044).

** Collegiate Runners (41.35 ± 6.53 mg/day) had a significantly greater iron intake compared to Collegiate Powerlifters (4.50 ± 6.53 mg/day) (*p* = 0.024).

**Table 4:** Micronutrient supplementation among female and male masters athletes.

Micronutrient Supplement	Masters Athletes		
	Females	Males	Combined
Vitamin A (mcg RAE)^a^	1,077.42 ± 142.66	906.86 ± 159.19	992.14 ± 106.88
Vitamin C (mg)^a^	259.28 ± 57.11	292.71 ± 63.73	276.00 ± 42.79
Vitamin D (mcg)^a^	8.18 ± 0.87	7.56 ± 0.97	7.87 ± 0.65
Vitamin E (mg d-alpha tocopherol)^a^	11.32 ± 3.53	12.89 ± 3.94	12.11 ± 2.65
B-1 (Thiamin) (mg)^a^	1.94 ± 0.54	1.96 ± 0.60	1.95 ± 0.40
B-6 (mg)^a^	1.80 ± 0.32	1.73 ± 0.33	1.76 ± 0.24
B-12 (mcg)^a^	4.62 ± 0.80	4.83 ± 0.89	4.73 ± 0.60
Folate (mcg DFE)^a^	392.06 ± 54.85	332.31 ± 61.21	362.19 ± 41.09
Niacin (mg)^a^	22.62 ± 5.59	22.67 ± 6.24	22.64 ± 4.19
Riboflavin (mg)^a^	2.04 ± 0.54	2.08 ± 0.61	2.06 ± 0.41
Calcium (mg)^a^	494.09 ± 65.73*	187.89 ± 73.34	340.99 ± 49.24
Iron (mg)^a^	21.21 ± 3.14	11.88 ± 3.51	16.55 ± 2.35
Magnesium (mg)^a^	51.14 ± 7.79	58.48 ± 8.69	54.81 ± 5.83
Selenium (mcg)^a^	23.80 ± 5.50	13.48 ± 6.13	18.64 ± 4.12
Zinc (mg)^a^	12.29 ± 2.51	15.73 ± 2.69	14.01 ± 1.88

mcg, micrograms; RAE, retinol activity equivalents; mg, milligrams; DFE, dietary folate equivalents.

^a^ Data are presented as estimated marginal means ± standard error.

* Masters Female Athletes (494.09 ± 65.73 mg/day) had significantly greater calcium intakes compared to Masters Male Athletes (187.89 ± 73.34 mg/day) (*p* = 0.002).

**Table 5:** Masters athletes sport classification micronutrient supplementation.

Micronutrient Supplement	Masters Athletes (*n* = 154)										
	Sport Classification										
	GA (*n* = 25)	RN (*n* = 56)	TA (*n* = 21)	CF (*n* = 11)	RW (*n* = 26)	CY (*n* = 3)^b^	RT (*n* = 4)	PL (*n* = 2)^b^	FH (*n* = 1)^b^	IH (*n* = 3)	SW (*n* = 2)^b^
Vitamin A (mcg RAE)^a^	937.61 ± 161.59	909.05 ± 105.34	722.08 ± 169.44	1,057.02 ± 243.07	758.51 ± 152.56	144.28 ± 447.81	793.57 ± 447.81	1,515.00 ± 548.45	1,515.00 ± 775.63	1,136.25 ± 474.97	2,056.07 ± 548.45
Vitamin C (mg)^a^	264.69 ± 64.69	197.60 ± 42.17	103.63 ± 67.83	350.71 ± 97.31	273.98 ± 61.08	77.14 ± 179.28	102.85 ± 179.28	560.00 ± 219.57	917.14 ± 310.52	348.57 ± 190.15	129.64 ± 219.57
Vitamin D (mcg)^a^	7.18 ± 0.99	6.54 ± 0.64	6.53 ± 1.03	8.07 ± 1.49	6.42 ± 0.93	2.61 ± 2.74	7.14 ± 2.74	12.50 ± 3.36	15.00 ± 4.75	8.39 ± 2.91	11.07 ± 3.36
Vitamin E (mg d-alpha tocopherol)^a^	10.54 ± 4.00	10.15 ± 2.61	6.31 ± 4.20	18.15 ± 6.02	21.86 ± 3.78	1.28 ± 11.06	7.07 ± 11.10	13.50 ± 13.60	13.50 ± 19.23	13.71 ± 11.78	14.07 ± 13.60
B-1 (mg)^a^	2.41 ± 0.61	2.26 ± 0.40	0.71 ± 0.64	3.52 ± 0.92	2.42 ± 0.58	0.14 ± 1.71	0.78 ± 1.71	1.50 ± 2.09	1.50 ± 2.96	2.91 ± 1.81	1.98 ± 2.09
B-6 (mg)^a^	1.95 ± 0.36	1.87 ± 0.24	0.95 ± 0.38	2.45 ± 0.55	1.81 ± 0.34	0.19 ± 1.02	1.04 ± 1.02	2.00 ± 1.24	2.00 ± 1.76	2.39 ± 1.08	2.66 ± 1.24
B-12 (mcg)^a^	4.56 ± 0.91	4.69 ± 0.59	2.81 ± 0.95	6.09 ± 1.37	4.81 ± 0.86	0.57 ± 2.52	3.14 ± 2.52	6.00 ± 3.09	6.00 ± 4.38	6.64 ± 2.68	6.44 ± 3.09
Folate (mcg DFE)^a^	351.38 ± 62.13	346.81 ± 40.50	193.50 ± 65.15	387.75 ± 93.46	292.17 ± 58.66	38.09 ± 172.19	476.18 ± 172.19	400.00 ± 210.89	400.00 ± 298.24	371.43 ± 182.63	842.85 ± 210.89
Niacin (mg)^a^	27.00 ± 6.34	25.46 ± 4.13	9.50 ± 6.64	37.87 ± 9.53	26.62 ± 5.98	1.90 ± 17.57	10.47 ± 17.57	20.00 ± 21.51	20.00 ± 30.43	32.85 ± 18.63	26.14 ± 21.51
Riboflavin (mg)^a^	2.50 ± 0.62	2.35 ± 0.40	0.86 ± 0.65	3.60 ± 0.93	2.51 ± 0.58	0.16 ± 1.72	0.88 ± 1.72	1.70 ± 2.11	1.70 ± 2.98	3.06 ± 1.82	2.15 ± 2.11
Calcium (mg)^a^	261.74 ± 74.45	247.60 ± 48.53	255.68 ± 78.07	319.18 ± 111.99	255.20 ± 70.29	22.85 ± 206.32	292.38 ± 206.32	240.00 ± 252.69	1,240.00 ± 357.36	322.85 ± 218.83	725.71 ± 252.69
Iron (mg)^a^	13.97 ± 3.56	17.74 ± 2.32	11.95 ± 3.73	15.93 ± 5.36	9.10 ± 3.36	1.71 ± 9.88	12.52 ± 9.88	18.00 ± 12.10	18.00 ± 17.11	18.14 ± 10.48	61.43 ± 12.10*
Magnesium (mg)^a^	42.95 ± 8.82	43.36 ± 5.75	46.23 ± 9.25	41.32 ± 13.28	44.47 ± 8.33	9.52 ± 24.46	52.38 ± 24.46	100.00 ± 29.96	100.00 ± 42.36	75.00 ± 25.94	85.71 ± 29.96
Selenium (mcg)^a^	20.67 ± 6.23	23.56 ± 4.06	10.10 ± 6.53	31.12 ± 9.37	12.36 ± 5.88	1.90 ± 17.26	10.47 ± 17.26	20.00 ± 21.14	20.00 ± 29.90	15.00 ± 18.31	47.14 ± 21.14
Zinc (mg)^a^	9.57 ± 2.84	10.67 ± 1.82	10.24 ± 2.98	23.84 ± 4.27	9.33 ± 2.68	1.43 ± 7.88	10.23 ± 7.88	47.14 ± 9.65**	15.00 ± 13.65	11.25 ± 8.35	18.35 ± 9.65

GA, general athlete; RN, runner; TA, triathlete; CF, crossFit; RW, rower; CY, cyclist; RT, resistance training; PL, powerlifter; FH, field hockey; IH, ice hockey; SW, swimming; mcg, microgram; mg, milligram; RAE, retinol activity equivalents; DFE, dietary folate equivalents.

^a^ Data are presented as estimated marginal means ± standard error.

^b^ Based on modified population marginal means.

* Masters Swimmers (61.43 ± 12.10 mg/day) had significantly greater iron intakes compared to Masters General Athletes (13.97 ± 3.56 mg/day) (*p* = 0.014), Masters Runners (17.74 ± 2.32 mg/day) (*p* = 0.03), Masters Triathletes (11.95 ± 3.73 mg/day)(*p* = 0.008), Masters CrossFit Athletes (15.93 ± 5.36 mg/day) (*p* = 0.043), Masters Rowers (9.10 ± 3.36 mg/day) (*p* = 0.003), and Masters Cyclists (1.71 ± 9.88 mg/day) (*p* = 0.011).

** Masters Powerlifters (47.14 ± 9.65 mg/day) had significantly greater zinc intakes compared to Masters General Athletes (9.57 ± 2.84 mg/day) (*p* = 0.015), Masters Runners (10.67 ± 1.85 mg/day) (*p* = 0.017), Masters Triathletes (10.24 ± 2.98 mg/day) (*p* = 0.020), Masters Rowers (9.33 ± 2.68 mg/day) (*p* = 0.013), and Masters Cyclists (1.43 ± 7.88 mg/day) (*p* = 0.019).

## 4. Discussion

The purpose of this study was to investigate supplement intake among Collegiate and Masters Athletes in various sport groups. As well as, examine sex differences in supplement intake among Collegiate Athletes and Masters Athletes. Our study is one of the first to compare micronutrient supplement intake among sex and multiple sport classifications in Collegiate and Masters Athletes, and also adds to the limited body of evidence on supplement use in Masters Athletes.

### 4.1. Collegiate athletes

When comparing sex, we reported that Collegiate male athletes had significantly greater vitamin A supplement intakes compared to Collegiate female athletes. The reported vitamin A supplement intakes in our study were higher than athletes of the same age in other studies (27, 28) Patlar et al. (27) supplemented 10 males, 22.85 ± 0.26 years of age, with 300 mg/day of vitamin A for four weeks. The participants completed a Bruce protocol test each week for four weeks. The authors (27) reported that vitamin A supplementation did not reduce lipid peroxidation and muscle damage post-exercise. Teixeira et al. (28) randomized 20 kayakers (6 females and 14 males), 19.1 ± 3.8 years of age (supplement group) and 20.3 ± 3.3 years of age (placebo group). The supplement group was given an antioxidant containing 272 mg of alpha-tochoperol, 400 mg of vitamin C, 30 mg of β-carotene, 2 mg of lutein, 400 mcg of selenium, 30 mg of zinc, and 600 mg of magnesium for four weeks. The athletes completed two 1,000-m flat water time trials in the four-week period. The authors (28) reported no significant differences in race time between either group pre- or post-supplementation. Lastly, since β-carotene was combined with other micronutrients, it is difficult to determine the direct effect of β-carotene. There is a lack of studies reporting the benefit of vitamin A or β-carotene on exercise performance (29–32). In their supplement consensus statements, neither the IOC or the International Society of Sports Nutrition (ISSN) list vitamin A as providing any performance benefit (30, 31).

Folate is a B vitamin responsible for red blood cell formation and amino acid metabolism. In our study, Collegiate female athletes' supplementation for folate (148.67 ± 67.50 mcg DFE/day) were lower than those reported by female athletes in previous studies (33, 34). Woolf et al. (33) reported higher folate supplementation among 58 highly active (564 ± 272 mcg DFE/day) and sedentary females (935 ± 438 mcg DFE/day) compared to our population. Bailey et al. (34) reported that folate intakes for females, 19 to 30 years of age, was 615 ± 17 mcg DFE/day, although they combined dietary and supplemental folate. Further, while most of the literature focuses on dietary intake of folate, there is limited evidence of folate supplementation intake among Collegiate female athletes. Inadequate folate intake for women puts them at risk for anemia, which may also affect their physical performance (4, 35).

We reported that Collegiate male athletes had significantly greater magnesium supplement intakes compared to Collegiate female athletes (Table 2). The reported intake of magnesium supplementation in our study was lower than athletes of the same age in other studies (36, 37). In a randomized control trial, Kass et al. (36) supplemented 16 male athletes, 20.88 ± 1.82 years of age, who had dietary magnesium intakes above and below the United Kingdom Reference Nutrient Intake (RNI) of 300 mg/day for adult males. The supplement group took 300 mg/day of magnesium oxide for two weeks. Participants in the placebo group did not receive supplementation. A maximal aerobic capacity cycle test was performed for 30 min, followed by three, five-second maximal isometric bench presses. The authors (36) reported no significant differences in aerobic and resistance performance between the supplement and placebo groups.

Cordova et al. (37) randomized 18 male cyclists, 26.2 ± 1.81 years of age, into magnesium-supplemented and control groups. The magnesium-supplemented group was given 400 mg/day (magnesium 400 supra Kapsel®, Sanct Bernhard, Barcelona, Spain), the control group did not receive a supplement. The aim was to examine the effect of magnesium supplementation in reducing muscle damage during a 21-day stage cycling race (“Vuelta a España”, which is about 3,300 km). The authors (37) reported no differences in performance between both groups.

While magnesium supplementation does not affect athletic performance (30, 31, 36, 37), it may be prudent for athletes to supplement in addition to their dietary intake to meet their respective Recommended Dietary Allowances (RDA). Volpe (38) stated that the majority of athletes are not meeting the RDA for magnesium. Further, Hunt et al. (39) reported that, although there is a high rate of magnesium supplement use among athletes, magnesium intakes are still inadequate to meet the average requirement.

We reported higher vitamin C supplementation in Collegiate CrossFit Athletes compared to other athletes; however, there is a lack of research on supplement in CrossFit Athletes (40, 41). Although, we reported similar vitamin C supplement intakes in Collegiate Athletes as other researchers (42–44); the current state of the evidence is not supportive of vitamin C being beneficial for athletes in general (45, 46). Merry and Ristow (47) state that, while antioxidants could play a role in recovery, vitamin C may reduce muscle hypertrophy signaling, in addition to hampering the athlete's ability to adapt to exercise training (31). For example, Paulsen et al. (43) supplemented 54 participants (28 females and 26 males), with vitamin C (1,000 mg/day) and vitamin E (235 mg/day) or a placebo for 11 weeks. The authors (43) reported that the combined vitamin C and vitamin E supplement did not improve maximal oxygen consumption (VO_2_max) or endurance performance after an 11-week endurance training program. Further, Paulsen et al. (44) aimed to observe if vitamin C (1,000 mg/day) and vitamin E (235 mg/day) supplementation would affect muscle growth and strength development during 11 weeks of supervised resistance training. Thirty-two resistance-trained females and males (20–45 years of age) were randomized into either a supplementor placebo group. The authors (44) reported equal increases in muscle hypertrophy between the supplemented and placebo groups. In addition, the vitamin C and vitamin E supplements interfered with protein synthesis and recovery.

In our study, we reported that Collegiate Runners had a significantly greater iron supplement intake compared to Collegiate Powerlifters. Iron supplementation has been shown to improve aerobic capacity in athletes with iron deficiency anemia (48, 49), and may be beneficial for athletes who are iron-deficient but nonameic (IDNA) (ferritin concentrations below 20 µg/L) (50, 51). The Collegiate Runner's iron supplement intake of 41.35 ± 6.53 mg/day, is similar to iron supplement intakes reported by other researchers (49, 50). For example, Pasricha et al. (49) reported iron supplementation doses ranging from 10 to 325 mg/day, for 4–24 weeks among 911 females (<18–50 years of age), 464 were given iron supplements. The authors (49) reported that iron supplementation improved exercise performance in females with iron deficiency. Similarly, Burden et al. (50) reported iron supplementation doses of one to three times per week, ranging from 10 to 425 mg/day for 6–15 weeks, in a meta-analysis on endurance athletes with IDNA. The meta-analysis included 17 studies with 443 participants (363 females and 80 males), 22.3 ± 5.1 years of age in which the researchers examined the effect of iron supplementation on VO_2_max. The authors (50) stated that VO_2_max improved with iron supplementation among athletes with IDNA. Finally, iron supplementation does not benefit athletes with a normal iron status and may lead to iron overload (48, 52).

### 4.2. Masters athletes

While calcium plays an important role in muscle contraction, supplementation is helpful when athletes' diets are deficient in calcium-rich foods or if they are restricting energy intake (30, 31). There is a lack of evidence on calcium supplementation's effects on athletic performance (53). Masters female athletes, took significantly more calcium supplements than Masters male athletes. We reported lower calcium supplement intakes, 494.09 ± 65.73 mg/day (females) and 187.89 ± 77.23 mg/day (males) than other researchers (54–59). Shea et al. (56) supplemented 33 females (61 ± 4 years of age) with a 1,000 mg/day of calcium or a placebo either before or during one-hour of walking. The authors (56) reported that calcium supplementation before and during exercises mitigates the loss of calcium during exercise. Similarly, Barry et al. (54) supplemented 22 male cyclists (37 ± 7.6 years of age) with a 1,000 mg/day of a calcium-fortified beverage or a control beverage either before or during a 35-km time trial. The authors (54) reported that calcium supplementation did not improve time trail performance compared to the placebo. Furthermore, calcium supplementation mitigated the increase in exercise-induced parathyroid hormone, but did not affect bone resorption activity.

We reported that Masters Swimmers, had significantly higher iron intake compared to Masters General Athletes, Masters Runners, Masters Triathletes, Masters CrossFit Athletes, Masters Rowers, and Masters Cyclists. Masters Swimmers' iron supplement intake, (61.43 ± 12.10 mg/day) aligns with other researchers (19, 60). Beshgetoor and Nichols (19) reported iron supplement intakes of 43 ± 8 mg/day among Masters Athletes (25 females, 52.5 ± 2 years of age). Further, the authors (19) analyzed four-day diet records and observed that Masters Athletes who supplemented with iron actually met their daily iron intake from diet al.one. McCormick et al. (60) supplemented 31 athletes (22 females and 9 males), 20–32 years of age, with 325 mg of ferrous sulfate and 500 mg of ascorbic acid, (105 mg of elemental iron) either daily or on alternating days for eight weeks. The athletes tracked their training data (minutes, kilometers, ratings of perceived exertion), which was converted into a total training load. The authors (60) reported that daily and alternate-day supplementation increased serum iron concentrations equally; and there was no mention of performance differences between the groups. Iron has not been shown to have any performance-enhancing benefits in athletes with normal iron status, although iron has been reported to improve aerobic performance in athletes with suboptimal iron status (49, 61).

We reported a higher intake of zinc supplementation in Masters Powerlifters (47.14 ± 9.65 mg/day) than other Masters Athletes, which is inconsistent to what others have reported (62, 63). Heffernan et al. (53) noted that zinc supplementation was popular with athletes due to zinc's potential to increase testosterone concentrations, yet the evidence is limited. For example, Shafiei et al. (62) assessed how exhaustive exercise affects testosterone concentrations in cyclists who were supplemented with zinc and selenium. The 32 male cyclists were assigned to one of four groups: 30 mg/day of zinc, 200 μg/day of selenium, 30 mg/day of zinc plus 200 μg/day of selenium, or a placebo for four weeks.

The cyclists performed pre- and post-supplementation exercise testing, consisting of two-minute cycling periods, alternating between 90% and 50% of the participants maximal workload. Zinc supplementation did not significantly increase resting testosterone concentrations compared to the other groups, when all groups consumed a diet sufficient in zinc (62). Conversely, Cinar et al. (63) supplemented 40 males (18–24 years of age) with 2.5–3 mg/g/day of zinc for six weeks. There were four groups: a sedentary group (control), a sedentary group with zinc supplementation, a zinc supplementation plus resistance training group, who resistance trained four times per week, and a resistance training group (with no supplementation). The authors (63) reported that testosterone concentrations increased in all groups, except for the control group. Although, high zinc supplementation has been shown to increase testosterone in sedentary and active young men (63), the use of high dose zinc supplementation can interfere with copper absorption, causing a copper deficiency (64). Additionally, zinc supplementation is used to support the immune system after strenuous physical activity, yet the International Olympic Committee (IOC) states that there is low-moderate evidence that zinc is beneficial for immune support; alternatively, a high intake of zinc supplementation may suppress the immune system (30).

### 4.3. Limitations

This was a cross-sectional study, only analyzing the data at a single point in time. The Block 2005 FFQ questions about supplements are limited only to single vitamin, mineral, and multivitamin and multimineral use. Additionally, there were no questions about protein powders, sport gels/powders, pre-workout supplements, fruit and vegetable powders. We did not survey the athletes to understand why they used supplements or where they obtained their information to decide on whether to supplement or not. Further, the small sample sizes in the Collegiate and Masters Athlete sport classification groups may have affected the differences in micronutrient supplementation within and between the groups.

### 4.4. Conclusion

While there is extensive evidence on the knowledge about the prevalence of micronutrient supplementation among Collegiate (6, 14, 22, 65, 66) and Masters Athletes (11, 20, 67, 68), it is also important to know about the dose, frequency, duration, and other patterns of supplement use. Our study is significant because there is limited research on the analyses and patterns of micronutrient supplementation among Collegiate and Masters Athletes in various sporting groups. Because dietary supplements are frequently used by athletes, our study adds to the sparse literature on Masters Athletes and their supplementation use. The findings from our research are beneficial for understanding the patterns of micronutrient supplementation among Masters Athletes. Additionally, understanding how dietary supplements are consumed by Masters Athletes in different sports can provide insight into how to improve the performance of these athletes. The results of our study could have a positive impact on enhancing the overall performance of Masters Athletes. In addition, our research might help to increase the knowledge surrounding dietary supplement use among Masters Athletes. Further research is needed to continue evaluating supplement micronutrient intakes among different types of Collegiate and Masters Athletes. Collegiate and Masters Athletes should consult qualified nutrition professionals about supplement use. Further research should examine micronutrient supplement plus dietary micronutrient intake among Collegiate and Masters Athletes to examine the extent that athletes exceed the Recommended Dietary Allowances, and the potential effects on health and performance.

## Data Availability

The raw data supporting the conclusions of this article will be made available by the authors, without undue reservation.
